# A wakeboarding injury presented as acute carpal syndrome and median nerve contusion after wrist strangulation: a case report

**DOI:** 10.1186/1757-1626-2-100

**Published:** 2009-01-29

**Authors:** Karsten Knobloch, Andreas Gohritz, Mehmed A Altintas, Marcus Spies, Peter M Vogt

**Affiliations:** 1 Plastic, Hand and Reconstructive Surgery, Hannover Medical School, Hannover, Germany

## Abstract

**Background:**

We present a case of combined median nerve contusion with immediate loss of sensation after the strangulation with a wakeboarding rope and prolonged referral to our department 72 hours after the injury accompanied by an acute carpal tunnel syndrome with immediate relief of numbness of a significant proportion of the median nerve following surgical decompression.

**Case presentation:**

The palmar branch of the median nerve was surrounded by a significant haematoma in addition to the strangulation damage caused by its more superficial location in contrast to the median nerve.

**Conclusion:**

In case of acute median neuropathy, urgent surgical intervention with exploration, decompression of both, the median nerve and the superficial branch of the median nerve, accompanied by compartment measurements of the forearm should be performed to regain or re-establish neurological integrity.

## Background

Wakeboarding began in the mid-1980s as a combination of waterskiing, surfing, and snowboarding. In the United States, an estimated 3.1 million people were wakeboarding in 2003 with increasing numbers. The wakeboarder or "rider" stands sideways on the wakeboard, similar to the stance used in snowboarding, and is pulled by a boat or an overhead cable system. The rider jumps over the wake of the boat, thus the name *wakeboarding*, and can perform various tricks, spins, or flips. Currently, only four papers deal with wakeboarding injuries [1-4].

A survey among 156 members of either the AOSSM or the Florida Orthopaedic Society in 2004 found that 15% had never heard of wakeboarding, while 36% of the orthopaedics questioned reported treating a wakeboarding injury during their daily-practice [2]. Those 57 Orthopedics treating 122 wakeboarding injuries found ACL tears and anterior shoulder dislocations as the predominant injuries. Fingertip avulsions were rare (2/122) as well as only a single fractured wrist (1/122). Among 60 wakeboarders (mean age 28 years, 47% intermediate, 25% advanced/professional wakeboarding expertise) in the United States with 82 injuries, only one fractured hand was encountered. Currently, no acute nerve compressions have been reported related to wakeboarding injuries. Acute nerve compression following water sport injuries has been reported in a single case concerning a bilateral peroneal palsy secondary to knee boarding behind a water ski boat [5].

While propeller injuries incurred in boating accidents have been reported in a case series of 32 injuries in a 15-year period [6], rope injuries in sports are rare. A facial trauma has been encountered following a snapped-back water ski towbar in a 28-year-old woman spotting a water-skier from the back of a boat striking her right eye with a hyphema, ecchymosis of the right eyelids, a right cheek laceration and a displaced maxillary fracture of the right medial wall and floor of the orbit necessitating several ophthalmic surgical procedures [7].

We report on a case with an acute carpal syndrome with concomitant median nerve contusion and soft tissue laceration of the palmar wrist following rope strangulation in mechanical towing machine in a wakeboarding athlete.

## Case report

A 19-year-old male was admitted to the Department of Plastic, hand and reconstructive surgery due to persistant dysaesthesia of the median nerve-supplied area and a soft tissue laceration of the palmar wrist following a wakeboarding accident three days before. As an intermediate wakeboarding athlete starting to wakeboard one year ago, he was wakeboarding on the largest German artificial wakeboarding circled nylon cable system on a lake with a speed of 40 km/h, when the automatic nylon cable system stopped immediately due to an overlapping wire. The male wakeboarder dived in the lake and got struck in the loose nylon cable system with his trunk and the hand. He managed to get off with the trunk still caught with his wrist in the nylon cable, when the nylon cable system was activated by chance and he was pulled 1/4 of the radius of the lake with a speed of 40 km/h, suffering a strangulation of his wrist with immediate numbness of the median-nerve supplied area of the left hand. Furthermore, he had a laceration of the palmar aspect of the wrist covering 0.5 × 5 cm tissue size.

At admission in a rural hospital, the two-point discrimination was > 15 mm for the entire interdigital nerves N1–N7 of the left hand as sensory loss in the median-nerve supplied area. The ulnar nerve was found without any pathology. Furthermore, he could not perform a wrist flexion, while wrist extension was possible. The capillary refill was 1s for all five fingertips. No distinct pain in the snuff box area was evident on admission. The left elbow joint had full range of motion, supination and pronation was limited due to persistent pain at the wrist level. Resting on a plaster, he was transferred three days after the initial injury with persistent clinical lesion of the median nerve in our department.

**Figure 1:**
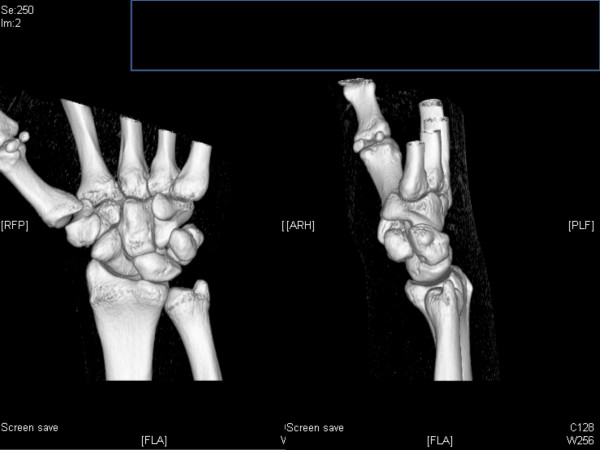
**Computer tomography of the wrist and the hand proofed regular bony structures**.

Conventional x-ray of the hand and the wrist found regular articulation without an evident bony lesion. No disruption of the scapular-lunar ligament was noted. Computer tomography of the wrist and the hand proofed regular bony structures (figures 1a, b). In the operating room dorsal compartment pressure of the forearm was 19 mmHg, at the palmar compartment of the flexor carpi ulnaris muscle 16 mmHg, which were both normal. The median nerve, 72 hours after the initial strangulation injury, appeared with hyperaemia and moderate swelling and limited haematoma in the carpal tunnel more according to a median nerve contusion. The palmar branch of the median nerve was surrounded by a significant haematoma, which was evacuated. The ulnar nerve was inspected and found without any significant signs of injury or haematoma.

The laceration area was excised completely and the skin could be closed primarily without compression with one subcutaneous drainage being inserted. On postoperative day 1, the patient regained the sensory function of the hand following 72 hours of acute carpal tunnel syndrome with median nerve contusion with remaining dysaesthesia of the thenar skin supplied by the palmar branch of the median nerve. On postoperative day 5 the patient was discharged home after an uneventful postoperative course. He complained of minor dysaesthesias in the mentioned thenar area with recurrent intensity and was advised to recovery for a total of four weeks before returning to sport.

## Discussion

Wakeboarding is a recent water sport with similarities to snowboarding regarding the board configuration and water ski as far as the pulling mechanism is concerned with mechanical cables or a boat pulling the wakeboarder. Mechanical cables as such can cause severe injuries [7], as seen in our case with a wrist strangulation with consecutive acute carpal syndrome, acute median nerve contusion and soft tissue laceration necessitating urgent surgical median nerve decompression and wound management.

Acute carpal tunnel syndrome is a rare entity. Following displaced physeal fractures of the distal radius among 109 children, two of them developed an acute carpal syndrome [8]. Volar displaced fragments of the distal radius are likely to cause acute carpal syndrome by decreasing the space in the carpal tunnel [9-11].

Acute median neuropathy after wrist trauma has been differentiated in acute carpal tunnel syndromes, necessitating urgent carpal tunnel release within 40 hours of the onset of the numbness and elevated carpal canal pressures greater than 40 mmHg from median nerve contusions, with normal carpal tunnel pressures, which are supposed to be treated by observation only [12]. In contrast, we present a case of combined median nerve contusion with immediate loss of sensation after the strangulation and prolonged referral to our department 72 hours after the injury accompanied by an acute carpal tunnel syndrome with immediate relief of a significant proportion of the median nerve following surgical decompression. We found only a minor haematoma within the carpal tunnel and no forearm compartment syndrome with normal compartment pressures. However, the palmar branch of the median nerve was surrounded by a significant haematoma in addition to the strangulation damage caused by its more superficial location in contrast to the median nerve.

Carpal fractures have been found in associated with acute carpal syndromes in case studies, such as after traumatic volar dislocation of the trapezoid [9,13], hamate and triquetral fracture [9,14,15] after scaphoid pseudarthrosis with concomitant rupture of the long flexor muscle tendon of the thumb, or scaphoid and 5^th ^metacarpal bone [16] as well as distal pole of the scaphoid and hamate fracture [14]. Among athletes, exertional carpal tunnel syndrome has been encountered among a golf player [18]. Acute carpal tunnel syndrome has been reported in a diver following decompression illness [17]. Among 3 or 22 drivers of the 1998 formula 1 World Championsship drivers [18] either exertional or chronic carpal tunnel syndrome has been complained. Wheelchair athletes are often encountered with chronic carpal tunnel syndrome [1,12] as well as rock climbers [21,22] and body building athletes [23].

In case of acute median neuropathy, urgent surgical intervention with exploration, decompression of the median nerve and the superficial branch of the median nerve, accompanied by compartment measurements of the forearm should be performed to regain or re-establish neurological integrity.

## Consent

The presented patient has approved and confirmed the presentation of the case report. Written informed consent was obtained from the patient for publication of this case report and accompanying images. A copy of the written consent is available for review by the Editor-in-Chief of this journal.

## Competing interests

The authors declare that they have no competing interests.

## Authors' contributions

All mentioned authors have participated substantially to the manuscript in both, conception and realisation of the case report. All have been involved in drafting of the manuscript.
